# Abortion learning mechanisms for nurses and midwives: a scoping review of evidence

**DOI:** 10.1080/26410397.2025.2518672

**Published:** 2025-06-27

**Authors:** Martha Nicholson, Lesley Hoggart

**Affiliations:** a PhD Researcher, The International Planned Parenthood Federation, London, UK; PhD Student, Faculty of Wellbeing, Education and Language Studies, The Open University, Milton Keynes, UK. *Correspondence*: Martha.nicholson@open.ac.uk; b Professor, Faculty of Wellbeing, Education and Language Studies, The Open University, Milton Keynes, UK

**Keywords:** abortion, scoping review, training, education, nursing, midwifery

## Abstract

Access to safe, affordable, and supported abortion care is a crucial component of reproductive justice and human rights. Abortion seekers consider nurses and midwives to be more supportive than other health professionals. Nurses and midwives have long been recommended providers of comprehensive abortion care, including second trimester care. This iterative scoping review aimed to explore the evidence on abortion learning mechanisms available to nurses and midwives and what can be improved about abortion training. Using the Arksey and O’Malley (Scoping studies: towards a methodological framework. Int J Soc Res Methodol. 2005;8(1): 19-32) and Levac et al. (Scoping studies: advancing the methodology. Implement Sci. 2010;5(1): 69) scoping review frameworks, four databases were searched, resulting in 879 articles published in English from 01.01.2010 to 01.08.2024. The authors included 43 studies and identified five learning mechanisms. The evidence is presented under three themes: (1) the adequacy of abortion learning mechanisms for nurses and midwives, (2) listening to nurses and midwives’ experiences, and (3) barriers to abortion training. This review found that in almost all legal and practice contexts, abortion training may be de-prioritised and hard to access because of institutional barriers, especially in centres of education. In conclusion, there is a low investment in abortion training for nurses and midwives. Policy-makers, health care systems, and educators should consider ways to continuously instil nurses and midwives with skills, confidence, and social authority to provide person-centred abortion care to combat harmful bias and mitigate the risk of reproductive coercion.

## Plain language summary

Abortion care should be safe, affordable, and available in all communities. To facilitate this, health professionals need information and confidence to provide abortion care. Nurses and midwives are the largest group of health professionals globally and can safely provide abortion care when trained. With this scoping review, we wanted to find out if abortion training for nurses and midwives is reported as adequate, and what can be improved. We searched for and reviewed journal articles published between 2010 and 2024. Firstly, we found that abortion training in nurses and midwives’ centres of education was reported as inadequate. However, abortion care training through “on-the-job” opportunities was more successful. Collaboration with external organisations was shown to be an optimal way to deliver abortion training. Secondly, we learnt that nurses and midwives feel frustrated and confused because of gaps in abortion knowledge. Where available, continuous training was shown to allow nurses and midwives time to develop confidence. This is especially important because, in many settings, abortion is criminalised and stigmatised. Lastly, nurses and midwives face significant barriers to learning about abortion care. Barriers include faith-based and personal views held by health professionals and educators, and the limited investment in abortion care training and provision. Overall, we found that nurses and midwives learn in diverse ways. However, even those working or learning in settings with supportive abortion laws may have inadequate abortion training. Nursing and midwifery educators should invest in training and collaborate with external organisations to boost knowledge and skills in abortion provision.

## Résumé

L’accès à des soins d’avortement sûrs, abordables et bénéficiant d’un appui est un élément essentiel de la justice reproductive et des droits humains. Les personnes souhaitant avorter considèrent que les infirmières et les sages-femmes les soutiennent davantage que d’autres professionnels de santé. Les infirmières et les sages-femmes sont depuis longtemps des prestataires recommandés pour des soins d’avortement complets, notamment au deuxième trimestre. Cette étude itérative de la portée visait à explorer les données sur les mécanismes d’apprentissage de l’avortement à la disposition des infirmières et des sages-femmes, ainsi que les améliorations possibles à apporter à cette formation. En utilisant les cadres d’étude de la portée d’Arksey et O’Malley (2005) et de Levac et al. (2010), quatre bases de données ont été consultées, ce qui a permis d’identifier 879 articles publiés en anglais entre le 1er janvier 2010 et le 1er août 2024. Les auteurs ont inclus 43 études et identifié cinq mécanismes d’apprentissage. Les données sont présentées sous trois thèmes: (1) l’adéquation des mécanismes d’apprentissage de l’avortement pour les infirmières et les sages-femmes; (2) l’écoute des expériences des infirmières et des sages-femmes; et (3) les obstacles à la formation à l’avortement. Cet examen a révélé que, dans presque tous les contextes juridiques et pratiques, la formation à l’avortement peut être dépriorisée et difficile d’accès en raison d’obstacles institutionnels, notamment dans les centres éducatifs. En conclusion, l’investissement dans la formation à l’avortement des infirmières et des sages-femmes est faible. Les décideurs politiques, les systèmes de santé et les éducateurs devraient envisager des moyens de transmettre de manière continue aux infirmières et aux sages-femmes les compétences, la confiance et l’autorité sociale nécessaires pour assurer des soins d’avortement centrés sur la personne, afin de lutter contre les préjugés néfastes et d’atténuer le risque de coercition reproductive.

## Resumen

El acceso a servicios de aborto seguros, asequibles y apoyados es un componente fundamental de la justicia reproductiva y los derechos humanos. Las personas que buscan un aborto creen que las enfermeras y parteras son más comprensivas que otros profesionales de salud. Desde hace mucho tiempo, se recomienda a las enfermeras y parteras como prestadores de servicios de atención integral al aborto, que incluye la atención en el segundo trimestre. El objetivo de esta revisión exploratoria iterativa era examinar la evidencia de los mecanismos de aprendizaje sobre aborto disponibles a enfermeras y parteras e identificar qué se puede mejorar en la capacitación sobre aborto. Utilizando los marcos de revisiones exploratorias de Arksey y O’Malley (2005) y Levac et al. (2010), se realizó una búsqueda en cuatro bases de datos, que produjo 879 artículos publicados en inglés de 01.01.2010 a 01.08.2024. Los autores incluyeron 43 estudios e identificaron cinco mecanismos de aprendizaje. La evidencia se presenta bajo tres temas: (1) la idoneidad de los mecanismos de aprendizaje para enfermeras y parteras, (2) escuchar las experiencias de enfermeras y parteras, y (3) barreras a la capacitación sobre aborto. Esta revisión encontró que en casi todos los contextos legislativos y de práctica, la capacitación sobre aborto podría ser despriorizada y difícil de acceder debido a las barreras institucionales, especialmente en centros de educación. En conclusión, se invierte poco en la capacitación sobre aborto para enfermeras y parteras. Los formuladores de políticas, sistemas de salud y educadores deberían considerar las maneras de infundir continuamente en las enfermeras y parteras habilidades, confianza y autoridad social para proporcionar servicios de aborto centrados en la persona, combatir los prejuicios dañinos y mitigar el riesgo de coerción reproductiva.

## Introduction

Access to safe, affordable, and supported abortion care is a crucial component of reproductive justice and human rights. Yet abortion services remain contentious in many settings today, causing confusion, prejudice, and a risk of reproductive coercion in health systems.^1,2^ Nurses and midwives can be equipped with knowledge and guidance to support abortion seekers through the continuum of comprehensive abortion care, from when a pregnancy is diagnosed through to counselling on options, medication, performing procedures and offering aftercare to those who want or need it.^3^ In many settings, nurses or midwives are likely to lead or assist with facilitating induced abortion via medication, a method that involves taking a combination regimen of misoprostol and mifepristone pills, or the misoprostol-only regimen where mifepristone is not available. Nurses or midwives may also lead or assist with in-facility procedures using manual vacuum aspiration (MVA) to remove the pregnancy.^4^

Research shows that nurses and midwives are as safe and acceptable providers of both methods of induced abortion as doctors are when given sufficient training.^5–7^ Women often consider abortion care from nurses and midwives as more supportive compared to other health professionals.^4^ Outside of direct abortion care provision, nurses and midwives may perform uncelebrated everyday tasks in abortion care. They take appointments, order supplies, accompany abortion seekers through protest lines, prepare equipment, and provide training or mentorship to colleagues.^6,8,9^

More generally, nursing and midwifery professionals play a crucial role in the realisation of universal health coverage through delivering a broad range of services in otherwise hard-to-reach communities.^10^ Including comprehensive abortion care in nurses and midwives’ scope of work has been a key objective on the WHO’s agenda for many years.^11^ Although nurses and midwives make up distinct occupational groups with context-specific roles and priorities, the WHO has found that similar issues and policy responses impact both professions.^12^ For instance, both nurses’ and midwives’ knowledges and roles can be undermined in health systems, so similar policy responses are needed to facilitate specialisation and leadership positions through education entry points and initiatives. Building on other reviews of nurses and midwives’ roles in abortion care,^6^ this review is interested in nurses and midwives’ exposure and experiences of preparing for a role in abortion care through learning.

In the light of positive research findings on the role and scope of nurses and midwives in abortion care, the WHO updated abortion guidance to include nurses and midwives as autonomous providers of abortion care, including second trimester in-clinic procedures.^4^ However, accessing and transferring knowledge on abortion in health care can be problematic due to bureaucratic blockages and exceptionalism,^13^ over-regulation of the nursing and midwifery roles in abortion care,^6,14^ and stigma-related barriers.^15–17^ Nurses and midwives may also be called upon to provide abortion care out of their scope in the absence of trained medical staff, especially in low-resource settings.^18,19^

There is substantial research on abortion training for medical staff and the inclusion or exclusion of abortion learning opportunities in doctors’ curricula.^17,20–25^ The inclusion of abortion care in nursing and midwifery curricula is comparatively understudied. However, researchers are increasingly calling attention to the gaps in nursing and midwifery training. For example, research with faculty members in Australian institutions highlights the institutional barriers to abortion training for nurses and midwives.^26,27^ Professional associations also point to a research gap on abortion learning in nursing and midwifery education.^28–31^

This scoping review aims to explore the evidence on abortion learning mechanisms available to nurses and midwives and what can be improved about abortion training.

## Methods

The lead author (MN) employed a systematic scoping review study design to enable a broad mapping of the evidence using the aim (to explore the evidence on abortion learning mechanisms and what can be improved about abortion training for nurses and midwives) to orient the search. MN used the Arksey and O’Malley 2005^32^ systematic scoping review framework to identify the search question, identify relevant studies, and to screen, chart and collate data.^32^ Using processes from the Levac et al.^33^ paper, MN then clarified and examined the purpose of this review in relation to the research aims, building in a continuous and iterative development of the charting process with support from her lead PhD supervisor (LH). Initially, the authors charted the data to identify the location, study aims, design, population group, and type of training. As the data charting process progressed, it became clear that nurses’ and midwives’ documented experiences of abortion learning and barriers to accessing and applying abortion knowledge are important foundations to these findings. Consequently, the authors also abstracted data on experiences of abortion training and factors that enable and prevent the delivery and application of such training.

The authors aligned their epistemological approach with Mak and Thomas^34^ to take a constructivist position and dispute the presence of an “absolute truth” in the findings that are presented here.^34^ Rather, the findings are constructed through examining the relationships between studies and the authors’ own understanding of abortion learning mechanisms. Regarding positionality, the authors brought an “outsider” perspective of the health system to this study, with non-clinical backgrounds. Their expertise in social sciences, public health and abortion stigma informed a broad and open conceptualisation of abortion training in this scoping review. MN is a PhD Candidate in health and well-being with a background in social anthropology and public health. LH is a professor of social policy research with expertise in abortion stigma. The authors sought to highlight the subjectivities of abortion knowledge and competencies, given the diversity of legal and cultural settings included in this review.

### Inclusion and exclusion criteria

The inclusion and exclusion criteria were developed iteratively. Peer-reviewed publications with the following eligibility criteria were included: (1) published between 1 January 2010 and 1 August 2024, (2) written or available in English, (3) reports on type of induced abortion care or post-abortion care training/education/capacity building/skills development/knowledge sharing/mentorship/task-sharing available to nurses and midwives, AND/OR factors that affect abortion training, education or task-sharing, (4) for a population of nurses and midwives, nursing or midwifery educators, nursing or midwifery students, or health system stakeholders,* (5) using any type of study design that involved primary research and (6) in any country setting. Table 1 presents an example of the search string employed for this review. The short publication range 2010–2024 was appropriate because of changes to abortion policy and legislation during this time. For example, in June 2022, the US Supreme Court overturned the *Roe v. Wade* decision, which had previously guaranteed nationwide access to abortion. Since 2022, abortion services have been heavily restricted in some states, which has inevitably affected the experiences and types of abortion training available. What’s more, 2010–2024 includes a period of research on abortion training for nurses and midwives that motivated changes to WHO guidance on professional roles in abortion provision. For example, a randomised control trial in Nepal assessed whether nurses and midwives could administer medical abortion pills as safely and acceptably as doctors.^35^ Publications were excluded if they (1) used study designs including review articles, commentaries, editorials, letters, case reports, protocols, historical or legal reviews, (2) only studied the effects of training interventions on patient populations, students’ knowledge, attitudes, and practices towards abortion, post-abortion contraception use, cost-effectiveness of abortion care or task-sharing, or (3) were specifically on miscarriage or pregnancy-loss.

**Table 1.** Search string for the PubMed journal database. Date of search: 5th August 2024

Abortion search terms	Population search terms	Education search terms	Learning mechanisms search terms
Abortion	Midwi* Nurs* Student midwi* Student nurs* Nursing student* Midwifery student*	Education Student College University	Train Task-shar* Competency Curriculum Learn*

(“abortion*” [Title]) AND (“midwi*”Title/Abstract] OR “nurs*”[Title/Abstract] OR “Student midwi*”[Title/Abstract] OR “Student nurs*”[Title/Abstract] OR “nursing student*”[Title/Abstract] OR “midwifery student*”[Title/Abstract]) AND (“education”[Title/Abstract] OR “student*”Title/Abstract] OR “college”[Title/Abstract] OR “university”[Title/Abstract] OR “train*”Title/Abstract] OR “task shar*”Title/Abstract] OR “competency”Title/Abstract] OR “curriculum”[Title/Abstract] OR “learn*”[Title/Abstract]) AND 2010/01/01:2024/01/01[Date – Publication].

### Databases and search strategy

Four databases were selected (PubMed, MEDLINE, PsycInfo, and SCOPUS) to facilitate access to studies published in nursing and midwifery journals, as well as journals covering public health, medicine, development studies, and social sciences. To organise reference lists, remove duplicates, screen abstracts, and full texts and extract data from studies, MN used the Covidence tool for literature reviews (Covidence systematic review software, Veritas Health Innovation, Melbourne, Australia. Available at www.covidence.org).

### Data charting process and analysis

Following a close reading of the 43 studies, we used the Covidence data charting function to extract data that responded to the aim: to explore the evidence on abortion learning mechanisms available to nurses and midwives and what abortion training can be improved. During the process, we added new fields, such as organisations offering the training, and removed others if deemed irrelevant. The national and/or regional abortion laws as well as policies for autonomous abortion provision by nurses and midwives were documented in Table 2. MN chartered and extracted the data independently, but consulted LH on the process throughout. After extracting the relevant data from all studies, MN exported the data into an Excel table and added comments on the facilitators and barriers presented to abortion learning in the studies, the links between the studies, and how the abortion learning related to general themes emerging from the data. The quality of studies was not assessed because it is not a standard protocol for scoping reviews and not required for identifying and mapping the evidence on a particular topic.^32^

**Table 2.** Abortion laws and provision policies among countries included in scoping review studies

Country (alphabetically)	Study authors	Abortion law at the time of publishing^1^	Nurses and/or midwives permitted to autonomously provide abortion drugs and procedures?^2^	
			Nurse	Midwife
Australia	Downing 2023 Mainey 2024	Available on request in most Australian states, but accessibility and gestational limit varies.	Varies by jurisdiction	Varies by jurisdiction
Australia (state of Victoria)	De Moel-Mandel 2019 Hulme-Chambers 2018	Victoria decriminalised abortion in 2008 and has a gestational limit of 24 weeks.	Yes	No
Brazil	Schroeter 2019	Permitted to save a person’s life and in cases of rape	Not specified	Not specified
Canada	Carson 2023 Dowler 2020 Sheinfeld 2016	Available on request with no legal restrictions, but in practice, accessibility and gestational limit vary at the provincial level.	Varies by province	Varies by province
Colombia	Vivas 2020	Broadly legal, but with uneven access across the country	No	No
DRC	Bourret 2020	Permitted to preserve health (including mental health), and in cases of rape, incest, or foetal diagnosis.	Not specified	Not specified
Ethiopia	Assefa 2019 Fekadu 2022	Broadly legal on social or economic grounds, and in cases of cases of rape, incest, or foetal diagnosis.	Yes	Yes
Gabon	NdembiNdembi 2019	Permitted to save a person’s life and in cases of rape, incest, or foetal diagnosis.	Not specified	Not specified
Ghana	Aborigo 2020 Chavkin 2018 Rominski 2016 Andersen Clark 2010	Permitted to preserve health (including mental health), and in cases of rape, incest, or foetal diagnosis	Not specified	Yes
Global	Fullerton 2018	n/a	n/a	n/a
Ireland	Stifani 2022	Available on request, up to a gestational limit of 12 weeks	Not specified	Not specified
Italy	Mauri 2017	Available on request, up to a gestational limit of 12 weeks	Not specified	Not specified
Japan	Mizuno 2014	Broadly legal on social or economic grounds	Not specified	Not specified
Malawi	Chakhame 2022	Permitted to save a person’s life	No data	No data
Nepal	Andersen 2016	Available on request, up to a gestational limit of 12 weeks	Yes	Yes (specified as senior auxiliary nurse midwives)
New Zealand	Whiting 2022	Available on request, up to a gestational limit of 20 weeks	Not specified	Not specified
Nigeria	Akiode 2010	Permitted to save a person’s life	Not specified	Not specified
Rwanda	Nkurunziza 2024	Broadly legal on social or economic grounds	Yes	Not specified
Somalia	Yalahow 2017	Permitted to save a person’s life	No data	No data
South Africa	Mpeli 2015	Available on request, up to a gestational limit of 12 weeks	Yes	Yes
Sweden	Endler 2020	Available on request, up to a gestational limit of 18 weeks	Not specified	Not specified
Tunisia	Hajri 2020	Available on request, up to a gestational limit of 12 weeks	Not specified	Not specified
Uganda	Paul 2014	Permitted to save a person’s life	Not specified	Not specified
England	Myerscough 2020	Broadly legal on social or economic grounds	No (approval from two doctors required)	No (approval from two doctors required)
United States (California)	Battistelli 2018 Freedman 2014 Levi 2018 McLemore 2015	Broadly legal on social or economic grounds	Yes	Yes
United States (Colorado)	Coleman-Minahan 2021 Coleman-Minahan 2020	Broadly legal on social or economic grounds	Yes	Yes
United States (Massachusetts)	Weintraub 2023	Broadly legal on social or economic grounds	Not specified	Not specified
United States (New York)	K. McNamara 2023	Broadly legal on social or economic grounds	Not specified	Not specified
United States (nationwide)	Bennett 2020 Simmonds 2019	Varies by State, from states that permit abortion to save a person’s life to states where abortion is available upon request with no gestational limit	Varies by state	Varies by state
Total	36			

^1^ The World's Abortion Laws – Center for Reproductive Rights (https://reproductiverights.org/maps/worlds-abortion-laws/?country=COD).

^ 2^ Global abortion policies database.

## Results

### Descriptive overview

**Figure 1.**
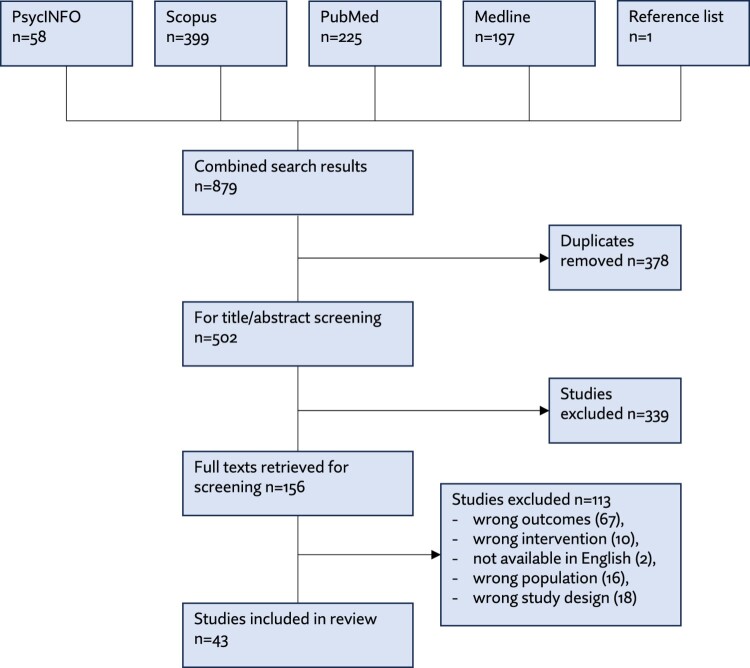
Screening flow chart

A search in August 2024 generated 879 papers from four databases: 378 duplicates were removed, 502 titles and abstracts were screened against the inclusion and exclusion criteria, and 163 full texts were retrieved. Of these, 113 studies were excluded using the criteria, and 43 were included in this review (see Figure 1).

**Figure 2.**
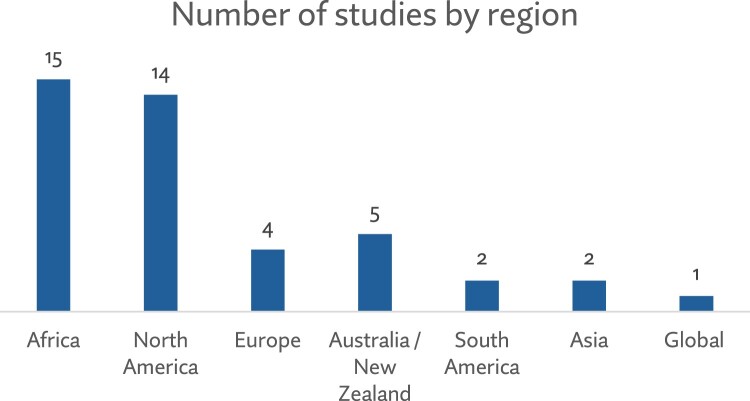
Number of studies by region.

**Figure 3.**
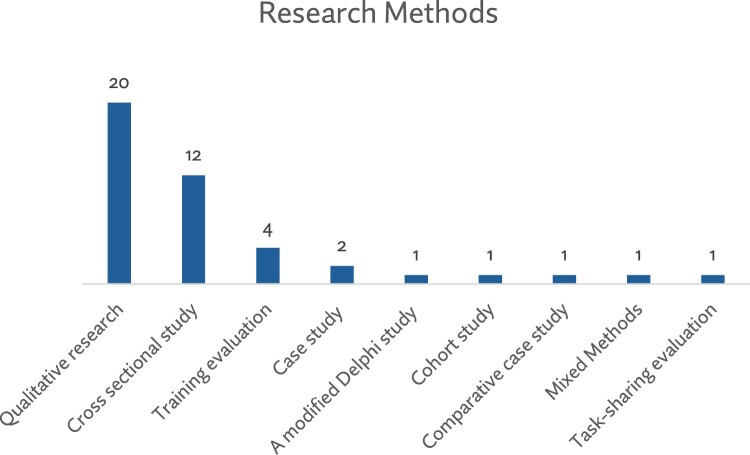
Research methods

**Figure 4.**
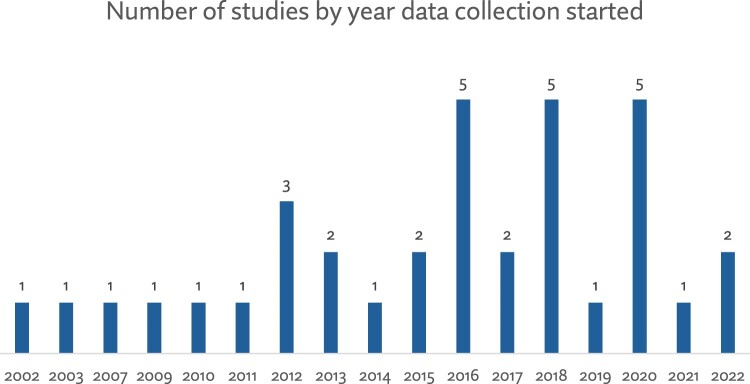
Number of studies by year of data collection

Out of the 43 papers included in this review, most were studies with populations in the African (*n* = 15) and North American (*n* = 14) regions (see Figure 2). Qualitative studies were the most common type of research design (*n* = 20) for presenting evidence on abortion training for nurses and midwives. These included focus group discussions, interviews, and case studies. Other studies used quantitative methods, mainly cross-sectional surveys – for instance, to assess nursing and midwifery students’ interest in abortion training (see Figure 3). Although all papers were published from 2010 to 2024, data collection for these studies commenced in 2002, as shown in Figure 4.

The scope and roles of nurses and midwives included in the studies differed, as well as the laws governing abortion care in the study settings. Table 2 presents the geographic scope and range of legal contexts for autonomous provision of abortion by nurses and midwives (if available) at the time of publishing. All studies included in this review explored training, mentoring, or educating nurses and/or midwives on abortion care. Studies presented findings from clinical, informal learning environments, and educational settings.

The literature included five categories of abortion learning mechanisms, as presented in Table 3. The most common forms of abortion training for nurses and midwives were supported practice through task-sharing programmes (*n* = 17), abortion content in student curriculums and/or didactic learning (*n* = 16), clinical training (*n* = 15), mentorship, informal learning, or provider support networks (*n* = 7), and value clarification workshops (*n* = 4).

**Table 3.** Abortion learning mechanisms

Mechanisms of learning about abortion for nurses and midwives	N of studies	Studies
Task-sharing programmes	17	Aborigo et al^36^; Andersen et al^18^; Bourret et al^19^; Andrea Carson et al^3^; A. Carson et al^37^; Chakhame et al^38^; Chavkin et al^39^; Dowler et al^40^; Endler et al^41^; Freedman & Levi^42^; Hajri & Belhadj^43^; Hulme-Chambers et al^44^; Mcnamara et al^45^; Paul et al^46^; Stifani et al^47^ ; Vivas & Valencia^48^; Weintraub et al^49^
Included in nursing and/or midwifery student curriculum and/or didactic training	16	Akiode et al^50^; Andersen et al^18^; Assefa^51^; Bennett et al^52^; Clark et al^52^; Coleman-Minahan et al^54^; Downing et al^26^; Endler et al^41^; Fullerton et al^55^; Mainey et al^27^; Mizuno^56^; Myerscough & Briscoe^57^; Nkurunziza et al^57^; Sheinfeld et al^59^; Simmonds et al^60^; Yalahow et al^61^
Clinical training	15	Akiode et al^50^; Andersen et al^18^; Bennett et al^52^; Bourret et al^19^; Chavkin et al^39^; Clark et al^53^; de Moel-Mandel et al^62^; Downing et al^26^; Freedman & Levi^42^; Levi et al^63^; Myerscough & Briscoe^57^; Nkurunziza et al^58^; Rominski et al^64^; Simmonds et al^60^; Whiting et al^65^
On-the-job mentorship, informal learning, and provider support networks	7	Andersen et al^18^; Bourret et al^19^; Andrea Carson et al^3^; A. Carson et al^37^; Freedman & Levi^42^; Mauri & Squillace^66^; McLemore et al^8^
Value clarification and attitude transformation (VCAT) workshops	4	Andersen et al^18^; Chavkin et al^39^; Mpeli & Botma^67^; Stifani et al^47^

Three major themes emerged from this analysis: (1) adequacy of learning mechanisms, (2) listening to learners, and (3) motivational and structural barriers to learning. These themes are further broken down into sub-themes. The first theme explores pre-registration training, embedded training in health systems and training in collaboration with external organisations. The second theme looks at nurses’ and midwives’ experiences of coping with inadequate abortion training, abortion training as a continuous process with provider networks, and mentors and pathways to confidence in abortion care. The final theme synthesises barriers to abortion learning in the literature.

### Theme 1 – adequacy of learning mechanisms

#### (In)adequacy of pre-registration training and education

Many papers posited that classroom-based learning and clinical training can normalise abortion and help students develop confidence and skills in empathy to support and care for abortion seekers.^57^ Training that exposes students to the everyday reality of abortion care can adjust preconceived ideas about what abortion may involve and shift narratives around abortion care from negative to positive.^57^ Nurses and midwives can be trained to be capable and acceptable providers of aspiration abortion, which is technically similar to intrauterine device insertion and endometrial biopsy.^42^

However, studies report that undergraduate nursing and midwifery modules rarely include adequate classroom-based training on comprehensive abortion care. Rather, piecemeal information about abortion care may be provided, if at all. For instance, a US study with nursing practitioner programme leaders reported that only 10% of primary care programmes offered didactic training on abortion procedures.^60^ The nursing and midwifery curriculum in Somalia, where abortion is highly restricted, is similarly unstandardised. Decisions on including abortion in the curricula were discretionary and influenced by educators’ sociocultural and religious beliefs. Newly graduated nurses in Rio de Janeiro, Brazil, where abortion is also restricted, felt unprepared to assist with abortion care under legal conditions, such as in the case of rape, because of minimal coverage of abortion care in their nursing education.^68^ Educators in Japan, where abortion is broadly legal, commonly taught only legal considerations, but rarely how to provide abortion procedures. Nursing faculty staff in Australia aspired to teach students competencies for preventing and caring for people with unintended pregnancies. In reality, few offered training on competencies, such as recognising the reproductive needs of vulnerable women (35.7%) or knowledge on types of abortion methods (16.7%).^26^ A knowledge, attitudes, and practices study of 405 nurses and midwives in Ethiopia, where abortion is also broadly legal, assessed the capacity of respondents to provide safe abortion care. Only 20.5% had received (unspecified) training on abortion care provision ^51^, indicating a similar shortfall in trained providers. These studies suggest that abortion laws do not determine the level of investment in abortion training in undergraduate learning.

Clinical training on MVA skills was rarely provided to nursing or midwifery students.^19,52,62,64,65^ Studies in Ghana, where abortion is legal to preserve health, reported that although many midwifery students receive classroom-based training in MVA, they did not simulate procedures in labs or have supervision in a clinical setting.^64^ In New Zealand and Australia, where abortion is broadly legal, survey studies found that there was a lack of training and professional supervision for nurses and midwives.^62,65^ A 2022 study from New Zealand highlighted a lack of educational advisory bodies to oversee abortion training and limited support from nursing and midwifery colleges.^65^ A comparative case study in the Democratic Republic of Congo (DRC), where abortion is permitted to preserve health, reported that MVA training for 350 midwives resulted in very few providing abortion or post-abortion care services due to similar challenging institutional and cultural norms in their workplaces.^19^ The minority of midwives who deviated from these norms to provide MVA services post-training cited having equipment to hand, professional support and being in a clinically urgent situation to which they could respond with MVA skills.^19^

The limited access to both clinical and didactic abortion training is shown to create confusion and unease amongst students. Student midwives in the UK, where abortion is broadly legal, found that only 4.5% of students had been taught about abortion during their studies, although some had accessed training before (3.6%). Amongst those who had received some superficial abortion training (“not in-depth, only brief”), they reported interchangeable terminology and confusion in rhetoric, causing vague understandings of guidance.^57^ However, studies show that educators understand the need for abortion training and that nurses and midwives may become advocates for abortion services if given training in the necessary competencies. This shows that nursing and midwifery educators can defy the norms to provide training on abortion for nursing and midwifery students and advocate for their students to be leaders in reproductive health care.

#### Embedding abortion learning in health care systems

*“*Task-sharing”, a common form of learning within health systems, is used to upskill nurses and midwives to provide autonomous abortion care. Studies show that successful task-sharing requires multi-stakeholder actions; health professionals need a supportive environment and resources for training on procedures such as MVA. Responsibilities should be delegated from medical teams^36^, and policies to broaden the scope of nurses' and midwives' roles should be supported and monitored by the Department of Health.^3,36,48^

Studies showed that abortion task-sharing initiatives have been developed to address inadequate abortion access in rural and remote areas in Australia, Ghana, and Nepal.^18,36,44^ A descriptive study of delegating abortion care suggests that midwives can mitigate against racism-based disparities in reproductive health care by supporting patient autonomy and increasing access to marginalised communities.^49^ Task-sharing was adopted to combat low staffing levels of doctors in primary care facilities in Ghana and the US^36,45^ and successfully reduced costs and waiting times for abortion care in Sweden.^41^ In Nepal, where abortion is legal upon request, researchers found that task-sharing increased access to abortion according to the number of geographic sites and providers offering abortion care.^18^

Professional support networks among nurses and midwives working in the health system can distribute skills and knowledge on abortion provision amongst peers and colleagues. A study exploring the recruitment and retention of nurses in abortion care provision in California, discussed the lack of formal career pathways in abortion care. Instead, nurses engaged in activities to emphasise the legitimacy of abortion care and explored growth opportunities through independent learning, mentorship and clinical exposure.^8^ Studies in Canada reported how nurse practitioners (NPs) arrived in abortion care provision via support and mentorship from colleagues. They were exposed to abortion work, informal education on regulations and best practices, and networked with lab technicians. They reported gaining confidence from relationships with mentors and the capacity for troubleshooting complex issues.^3,37^ Midwives who provided second trimester abortion care in Italy reported that cooperation with colleagues was crucial for allocating abortion work and sharing knowledge and competencies. For these midwives, training did not end in formal education, but was a continuous process; “you will never be prepared enough”.^66^

#### Collaborations for abortion training

NGOs and civil society actors are shown to be strong providers of abortion training for nurses and midwives. For example, Ipas and the National Health Training Centre for Nepal collaboratively developed theoretical training on how to provide medical abortion. They focused on service delivery, client follow-up, risk management, and the special needs of marginalised groups. Evidence from an evaluation of a multi-pronged intervention showed that Ipas increased access to abortion by boosting the number of providers and service delivery sites.^18^ In the US, where abortion was broadly legal at the time of writing, NPs and certified nurse-midwives (CNMs) were recruited to health workforce pilot projects and trained to provide aspiration abortion based on competency-based medical education principles.^63^ A curriculum was developed, incorporating abortion care training guidance from medical textbooks and a professional toolkit for advanced practice clinicians on integrating abortion care into their practice. The researchers found that this initiative successfully prepared clinicians’ processes for attaining aspiration abortion skills.^63^

Participation in value clarification workshops led by external training providers, like NGOs, can help nurses and midwives to clarify their roles in abortion care and facilitate peer-to-peer learning.^18,39,47,67^ In Ireland, external trainers were invited to deliver value clarification workshops after abortion was legalised to help clinicians define their roles.^47^ Value clarification workshops for student midwives in South Africa, where abortion is broadly legal, were found to be transformational. They gave participants an opportunity to reflect and emotionally engage in abortion patients’ needs. In particular, a so-called “5-minute” session on empathising with the perspective of a marginalised person encouraged more tolerant views towards abortion patients. ^67^ NGOs in Ghana and Nepal delivered a trainer of the trainer model for clinician-focused value clarification for action and transformation (VCAT) workshops as part of the implementation of externally funded abortion programmes.^18,39^

### Theme 2 – Listening to nurses and midwives

The authors identified three subthemes on nurses and midwives’ experiences of learning about abortion care: (1) coping with inadequate abortion training, (2) abortion training as a continuous process with provider networks and mentors, and (3) confidence development in abortion service delivery.

#### Coping with inadequate abortion training

Nurses and midwives reported experiencing confusion, stress, and frustration when not given adequate information on abortion care.^37,57,65^ Several studies highlighted how nurses and midwives lack confidence in their knowledge and feel ill-equipped to respond to patients’ needs because of the limited availability of training. A study on pregnancy options counselling by nurse practitioners in rural Colorado found that “respondents do not feel adequately trained to provide abortion counselling, and perceived lack of knowledge is the most common reason for unwillingness and inability to counsel on abortion”.^69^ Midwifery students interviewed in the United Kingdom expressed confusion over the conflicting information they had picked up on abortion during training.^57^ Newly graduated nurses recruited to a study in Brazil, where abortion is permitted to save a person’s life, felt that their education was outdated, lacked a feminist perspective and meant that they were ill-equipped to provide services needed by women in their communities.^68^

Nursing and midwifery staff have demonstrated their coping mechanisms and abilities to fill the gaps in formal abortion guidance and training themselves. Midwives in Ireland reported how they struggled with (and managed) the implementation of a new abortion law without any training. For example, a quote from a study by Stifani et al. in 2022 read: “Literally, when I started in January, I got handed a folder with a bunch of information on all the legislation, on the consent forms and everything. So, I literally had to do a lot of reading on all the documents I had to see what the guidelines were. I kind of had to educate myself on that”.^47^

#### Abortion training as a continuous process with provider networks and mentors

Nurses and midwives reported in several studies that their capacity to provide abortion care improved when they had access to support networks and continuous development opportunities. For instance, Canadian NPs who participated in a formal mentorship scheme by the Society of Obstetrics and Gynaecology of Canada (SOGC) described it as highly motivating and supportive. The SOGC mentees were coached through new experiences in abortion care and received help with preparing for real-life practice after training.^37^

Elsewhere, provider support networks and feminist collectives play a similar role in delivering, supporting, and following up abortion training for nurses and midwives. For example, the Middle African Network for Women's Reproductive Health collaborated with midwifery training colleges to endorse midwives as MVA providers in Gabon, Cameroon, and Equatorial Guinea. This collaboration boosted midwives’ skills despite opposition from hospital administration staff.^70^ The Colombian feminist collective “La Mesa” helped health care workers advocate for abortion care pathways in hospitals at a time when legal restrictions prevented abortion unless there was a risk to a person’s life or health. La Mesa offered this championship of abortion in the health system in the context of no formal training or institutional support from universities or hospitals.^48^ These collaborations show how principles of feminist organising and solidarity can support and mentor nurses and midwives to expand the interpretation of abortion laws, develop abortion care pathways and provide care and support in local institutions. In the context of opposition to abortion, supportive experiences with mentors and allies in the health system can ensure nurses and midwives are able to champion abortion care.

#### Pathways to confidence in abortion service delivery

The criminality, stigma, and silence associated with abortion care make the pathway to confidence in abortion care provision especially challenging for nurses and midwives. Even senior midwives have been shown to need training on the basics of abortion care in previously restricted settings like Ireland because they lacked knowledge and confidence in the psychosocial, legal, and logistical aspects of abortion care.^47^

Another study showed how clinicians were able to develop confidence in abortion procedures in diverse and individual ways, depending on the contexts and experiences they carry with them. For instance, a study on MVA training and provision in the US showed how registered NPs, nurse midwives, or physician assistants learned to offer MVA through a combination of reading study materials, observing physician trainers performing procedures, providing procedures under close instruction, and finally providing procedures with trainers nearby but not in the room. The strategies varied according to the different learning types: vigilant, experiential and progressive confidence.^42^

Research on midwifery roles in MVA in the DRC found that confidence to provide abortion and post-abortion care may be developed through responding to urgent medical needs. Midwives reported that knowing MVA equipment was on hand and being supported by colleagues allowed them to confidently treat patients with post-abortion care. Once established as MVA providers, midwives were able to further leverage their confidence to teach and inspire their colleagues to provide MVA.^19^ These insights suggest that abortion training initiatives for nurses and midwives must be accompanied by the institutional conditions and opportunities to elevate their authority and confidence to lead others and provide abortion care. Since colleagues, educators, patients, and health system managers may oppose their roles in abortion care, nurses and midwives must overcome hurdles to be confident providers of abortion.

### Theme 3 – Barriers to abortion training

Barriers to abortion care training were a pervasive theme across all geographic and institutional levels. These constraints, summarised here under sub-themes (1) structural/contextual barriers and (2) motivational barriers, prevent nurses and midwives from acting on intentions to learn, apply knowledge and skills on abortion care in their work.

#### Structural/contextual barriers

Even where abortion education is provided, institutional resistance in the health system may not allow individual nurses and midwives to apply their knowledge. For instance, abortion training in Ghana did not improve the odds of midwives providing post-abortion care to patients in the health system because of institutional resistance from other health professionals.^53^ Similarly, in the US, a national study found that opposition to abortion was common in teaching hospitals and limited NPs’ ability to engage in clinical training. However, the institutional culture against abortion varied by region, showing that the hospital staff reflected the state policy environment.^52^ Educators in Australia reported avoiding abortion to “protect” students and colleagues from the “unmentionable” topic of abortion. To overcome these perceived barriers, some educators creatively disguised abortion learning as modules on global health to overcome the stigma.^27^

Limited investment in abortion learning presents a structural barrier to the flow of knowledge to nursing and midwifery students. For example, task-sharing post-abortion care with midwives in Uganda, where abortion is permitted to save a person’s life, was limited because of a lack of equipment and medication in district facilities.^46^ Nursing and midwifery educators in Australia revealed that training programmes were “already overcrowded” and that abortion slipped through the net because curricula mapping across professions was not done.^27^ Practical barriers to abortion learning in health systems were raised in the literature. For instance, midwifery and nursing educators in Canada, where abortion is fully decriminalised, rarely offered abortion education in the classroom because they reasoned that they did not expect students to encounter abortion during placements, so they did not put the topic forward as a priority.^59^ This highlights a negative feedback loop between abortion care capacity in the health system and the case for training nurses and midwives in centres of learning.

#### Motivational barriers

A lack of political will and ambivalence to abortion was reported amongst health professionals in Tunisia. This prevented medical teams from delegating abortion care responsibilities to midwives in a task-sharing effort.^43^ There was a similar ambivalence to abortion learning amongst nurses and midwives in Ghana who were not adequately recognised or remunerated for additional responsibilities and emotional labour.^39^ Midwives in New York hospitals resisted adopting responsibility for aspiration abortion care, proposing that it should be maintained in the scope of the practice of doctors.^45^ A study with educators on nursing and midwifery programmes in Rwanda revealed that 41% held anti-abortion personal and religious beliefs, and excluded abortion from their programmes.^58^ These studies suggest that nursing and midwifery educators may also require training on abortion to enhance their self-efficacy in abortion care provision and delivering education. A national scheme to improve post-abortion care training for midwifery instructors and students in Nigeria resulted in significant improvements to midwives’ provision of post-abortion care.^50^ Personal beliefs on abortion pervade the transfer and acceptance of theoretical and clinical knowledge for nursing and midwifery students.

## Discussion

This review aimed to explore the evidence on abortion learning mechanisms available to nurses and midwives and what could be improved about abortion training. We found there to be a range of abortion learning mechanisms reported in the literature, but also found that nurses and midwives have limited opportunities to access them, especially in centres of education. Evidence on abortion learning from diverse country settings, legal contexts, and institutions found that all too often, pre-registration students receive unclear guidance in their education, and registered professionals may face institutional resistance to learning about abortion in the health care systems where they work. Studies show that training programmes are overburdened, lack resources or have educators and student bodies that are too ambivalent to engage in abortion training.^27,39,43^ The key finding in almost all settings, irrespective of legal status, is that there is a ubiquitous lack of investment in nurses and midwives’ training on abortion care. This means that nurses and midwives may have inadequate or limited knowledge and skills on how to respond to pregnant people’s reproductive health needs.

These findings align with previous studies on nurses and midwives’ roles in abortion care to suggest that institutions “over-regulate” nurses and midwives, and do not endorse them as trusted knowers or providers of abortion care.^6^ In the context of optimistic calls for expanding abortion education for nurses and midwives from researchers and reproductive health advocates ^6,26,27,29,71^, from the WHO^4^ and elsewhere, this review presents troubling structural and motivational barriers to embedding knowledge on abortion into nurses' and midwives’ roles. Unlike the WHO guidance on nurses' and midwives' role published on an international level, this review finds there is a lack of clear and visible policies available on nurses' and midwives’ roles in abortion care on the national and institutional level, sowing confusion, and discriminatory practice in education.^57^ We have thus found ample evidence to support Millar’s^13^ argument that developing knowledge on abortion in health care is problematic due to bureaucratic blockages and exceptionalism.^13^

Although we know that pregnant people can self-manage their abortions safely outside of the health system^72^, nurses' and midwives’ capacity to provide abortion care is a crucial component of a safe and destigmatised abortion ecosystem.^4^ The limited abortion training reported in this review may be a threat to the practical implementation of abortion laws, even where legal ground for increasing accessibility has been gained.

Despite major challenges to abortion learning, nurses and midwives also reported positive experiences of training to provide abortion care. Training that was embedded into health systems through task-sharing and supportive networks were shown to allow for continuous training opportunities (theme 2.2) and to boost confidence (theme 2.3).^8,73^ These findings resonate with the results from a recently published randomised controlled longitudinal trial of value clarification training workshops, which reported that improvements in providers’ abortion knowledge and attitudes following participation diminish over time.^74^ This suggests that abortion training mechanisms in health systems may be most effective through supportive initiatives like mentorship, solidarity networks and provision of clear and actionable guidelines to maintain knowledge and pro-abortion attitudes over time. In this way, stigma-related barriers identified in research.^15–17^ may be challenged.

Given the findings on limited access to abortion training in centres of education, nursing and midwifery educators should consider the significant motivational and structural hurdles that nurses and midwives may face when negotiating their knowledge and abortion provision skills. Students should be appropriately equipped with opportunities to clarify their position on abortion and their roles. Where abortion training is absent from curricula or poorly provided, nurses and midwives may have to seek out inconsistent and confusing information about abortion laws and practice to fill the gaps in their training. This could be potentially harmful to patients who legitimately seek abortion care, and is problematic given that nurses and midwives are widely recommended as providers of abortion.^5–7^

### Limitations and strengths

MN conducted and wrote this review as part of a PhD study with support from LH and a university librarian. There is a risk that the search, which was conducted by one author, missed articles.^34^ Studies were also only included if published in English, which caused two studies to be excluded. The authors were not able to do a comparison of the evidence on abortion training for medical versus procedural abortion care, largely because studies did not always differentiate the abortion methods that students were trained on. Many presented learning mechanisms that covered a combination of abortion methods. A quality review of the studies was not in the remit of this scoping review, so the accuracy of findings was not assessed. Lastly, the abortion laws and policies listed in Table 1 were relevant for the time of publishing of the articles identified in this review and may, in some country contexts such as the US, be outdated upon publishing. However, the strength of this review is identifying what the research landscape for abortion learning mechanisms looks like now from a reflective standpoint. This review presents what we know about the experiences of abortion learning for nurses and midwives, but highlights the barriers they may face.

## Conclusion

Nurses and midwives may learn about abortion in diverse ways, but embedding continuous training in health systems and collaborating with external partners such as NGOs can boost their confidence to provide abortion services. Nurses and midwives face significant barriers to pre-registration abortion training, which institutions such as universities should urgently address, and health care systems should be aware of. Nurses and midwives can benefit from not only clinical or didactic training but also continuous coaching and mentoring to enhance their confidence and social authority to combat any harmful bias and discrimination they may encounter.

## Footnotes

* The professional roles of nurses and midwives take different forms and titles depending on the context. For this review, we included the following 1) advanced practice clinicians (APCs), 2) auxiliary nurse midwives (ANMs), 3) certified nurse midwives (CNMs), 4) midwives 5) nurses, and 6) nurse practitioners (NPs).

## Provenance statement

This article was not commissioned and went through external review.
